# HGBL-NOS presenting as widespread extranodal disease without lymphadenopathy: a case report

**DOI:** 10.3389/fonc.2026.1781511

**Published:** 2026-03-18

**Authors:** Mohammad Fuad Sayes, Ali Sabbah, Akram Karama, Ali Allan, Basel Bader, Mohannad Abulihya

**Affiliations:** 1 Al-Quds University, Jerusalem, Palestine; 2 Department of Hematology & Oncology, Istishari Arab Hospital, Ramallah, Palestine; 3 Department of Pathology, Istishari Arab Hospital, Ramallah, Palestine

**Keywords:** case report, central nervous system involvement, extranodal lymphoma, HGBL-NOS, high-grade B-cell lymphoma, lymph node-negative, R-CODOX-M/IVAC

## Abstract

High-grade B-cell lymphoma, not otherwise specified (HGBL-NOS), is a rare and highly aggressive B-cell lymphoma with overlapping morphologic and immunophenotypic features that complicate distinction from other aggressive B-cell lymphomas. Disseminated extranodal involvement, absence of lymphadenopathy, and lack of standardized treatment guidelines pose significant diagnostic and therapeutic challenges in HGBL-NOS. We report a 27-year-old female who presented with a six-month history of progressive left-sided chest pain, dyspnea, hemoptysis, and dry cough without constitutional B symptoms. Laboratory evaluation revealed anemia and markedly elevated lactate dehydrogenase (LDH) levels. Imaging revealed a large, heterogeneous mass in the anterior left lung with disseminated extranodal involvement of 11 organs, including the lungs, heart, brain, kidneys, adrenal glands, uterus, spleen, pancreas, bone, skeletal muscle, and thyroid, in the absence of lymphadenopathy. Endobronchial ultrasound-guided transbronchial (EBUS) biopsy confirmed infiltration by atypical large B-cell lymphoid cells. Immunohistochemistry revealed diffuse positivity for CD20, CD10, and BCL6, with C-MYC expression in 40% of tumor cells and a Ki-67 proliferation index of 90%. The patient received R-CEOP for cytoreduction, followed by four cycles of intensive R-CODOX-M/IVAC chemotherapy, with dose adjustment based on hepatic and cardiac tolerance. Post-treatment positron emission tomography/computed tomography (PET/CT) confirmed complete metabolic remission. This case highlights an exceptionally rare and aggressive presentation of HGBL-NOS with extensive extranodal dissemination involving 11 organs in the absence of lymphadenopathy, underscoring the importance of early integrated diagnostic approaches and prompt initiation of intensive chemotherapy to achieve favorable outcomes.

## Introduction

High-grade B-cell lymphomas (HGBL) are a subset of aggressive mature B-cell neoplasms that are characterized by rapid growth, aggressive clinical behavior, and a poor prognosis. HGBL exhibit intermediate features between diffuse large B-cell lymphoma (DLBCL) and Burkitt lymphoma (BL) (1).

According to the 2022 WHO 5th edition, HGBL are subdivided into three categories: Diffuse large B-cell lymphoma/high-grade B-cell lymphoma with MYC and BCL2 rearrangements, high-grade B-cell lymphoma with 11q aberrations, and high-grade B-cell lymphoma-NOS (2). In contrast, the International Consensus Classification (ICC) divided HGBL into two major categories: HGBL with MYC and BCL2 and/or BCL6 rearrangements, and HGBL-NOS (3).

HGBL-NOS is rare, accounting for approximately 1%-2% of non-Hodgkin lymphomas (4). The WHO emphasizes that it poses diagnostic challenges, and it is diagnosed only when pathologic findings cannot classify the case as DLBCL or BL (1).

The lack of prospective clinical trials on treatment of HGBL-NOS poses a significant therapeutic challenge, given that no standardized first-line treatment has been established (1). In this case, the patient received an Intensified R-CODOX-M/IVAC (Rituximab plus Cyclophosphamide, Vincristine, Doxorubicin, and high-dose Methotrexate alternating with Ifosfamide, Etoposide, and high-dose Cytarabine) chemotherapy regimen, chosen particularly due to the central nervous system (CNS) involvement (1, 5–8). This case is reported due to its highly unusual presentation, characterized by extensive extranodal involvement of 11 organs in the absence of lymphadenopathy. It provides clinically relevant diagnostic and therapeutic insights for managing HGBL-NOS.

## Case description

### Clinical presentation and initial evaluation

A 27-year-old woman who is gravida 1, para 1 (G1P1) with a 10-year history of smoking approximately two shishas per week, presented to the emergency department with a six-month history of progressive left-sided chest pain, dyspnea, hemoptysis, and dry cough. She denied constitutional “B symptoms,” including fever, night sweats, or unintentional weight loss, and reported no gastrointestinal or genitourinary complaints.

Her medical history was unremarkable, with no personal or family history of malignancy or autoimmune diseases and no prior exposure to chemotherapy or radiation.

On admission, vital signs revealed elevated blood pressure (144/97 mmHg) and tachycardia (102 beats per minute), while other parameters were within the normal limits. Physical examination demonstrated dullness to percussion over the left hemithorax between the 4th and 7th intercostal spaces with decreased breath sounds over the left mid-lung. No lymphadenopathy or hepatosplenomegaly was detected.

### Diagnostic assessment

#### Laboratory findings

Initial laboratory investigations revealed anemia, elevated inflammatory markers, abnormal liver function tests, and metabolic disturbances (**Table 1**).

**Table 1:** Summary of laboratory results at initial presentation.

Laboratory test	Result	Reference range
Hematology		
Hemoglobin (g/dL)	10*	12–16
Hematocrit (%)	32.1*	36–46
MCV (fL)	69.8*	80–100
RDW (%)	19*	11.5–14.5
Neutrophils (10³/µL)	9.05*	2–7
Eosinophils (%)	2	1–6
Basophils (%)	0.2	0–1
Chemistry/metabolic panel		
Albumin (g/dL)	3.36	3.5–5.0
Creatinine (mg/dL)	1.23	0.6–1.2
Sodium (mmol/L)	133*	135–145
Potassium (mmol/L)	3.98	3.5–5.1
Calcium (mg/dL)	7.66*	8.5–10.5
Liver function/enzymes		
AST (U/L)	183.7*	10–40
ALT (U/L)	371.5*	7–56
LDH (U/L)	537*	140–280
Inflammatory markers		
CRP (mg/L)	130.7*	<5
Coagulation		
PT (sec)	12.7	11–13.5
PTT (sec)	28.3	25–35
INR	0.9	0.8–1.2

Bold values indicates statistically significant and/or abnormal values.

### Imaging studies, histopathology and immunophenotyping

In Oct 2024, Chest radiography revealed a large, irregular, ill-defined, heterogeneous anterior left-sided mass measuring 13.3 × 5.2 cm, extending from the upper to the middle lung lobes, with a small pericardial effusion.

On 19 Oct 2024, subsequent thoraco-abdominal-pelvic computed tomography (CT) revealed bilateral renal enlargement with heterogeneous echotexture, adrenal gland thickening, hypoechoic pancreatic masses, and enlarged ovaries with increased vascularity.

**Figure 1:**
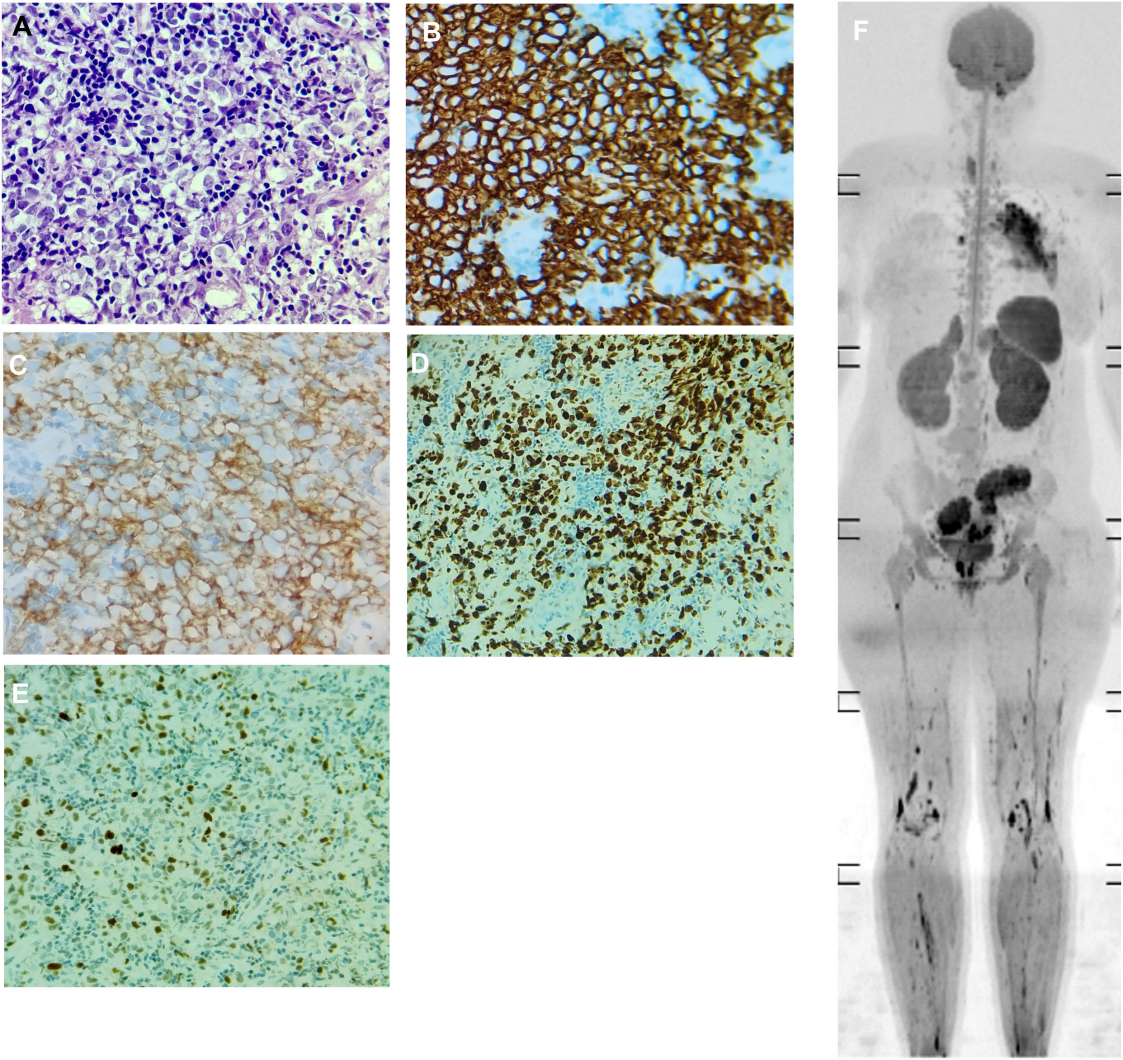
Histopathological, immunohistochemical, and imaging findings. **(A)** Hematoxylin and eosin-stained (H&E) section shows sheets of medium- to large-sized atypical lymphoid cells with irregular round to oval nuclei, fine chromatin, prominent nucleoli, and a moderate cytoplasm. The tumor exhibits frequent mitotic figures and scattered apoptotic bodies. No classic “starry sky” pattern typical of Burkitt lymphoma is observed. **(B)** CD20 immunostain demonstrates diffuse B-cell positivity, confirming B-lineage. **(C)** CD10 immunostain demonstrates diffuse positive expression consistent with germinal center B-cell (GCB) phenotype. **(D)** Ki-67 demonstrates a high proliferation index (~90%), supporting high-grade morphology. **(E)** C-MYC immunostain shows nuclear positivity supporting high-grade lymphoma features. **(F)** Whole-body diffusion-weighted MRI shows infiltrative lesions involving the ovaries, uterus, kidneys, adrenals, pancreas, spleen, brain, bones, muscles, thyroid, heart, and lungs.

On 3 Nov 2024, EBUS biopsy of the mass revealed dense infiltration of the bronchial wall by highly atypical large lymphoid cells. Immunohistochemistry (**Figures 1A–E**) showed diffuse positivity for CD20, CD10, and BCL6, with C-MYC expression in 40% of tumor cells and a Ki-67 proliferation index of 90%. Tumor cells were negative for CD3, BCL2, and MUM1. These findings were consistent with HGBL-NOS. Bronchoalveolar lavage revealed a predominance of neutrophils (55%), macrophages (40%), and lymphocytes (5%), without malignant cells or granulomas.

On 3 Nov 2024, Echocardiography revealed left ventricular ejection fraction (LVEF) of 55%, left ventricular hypertrophy with minimal pericardial effusion, consistent with myocardial infiltration.

**Figure 2:**
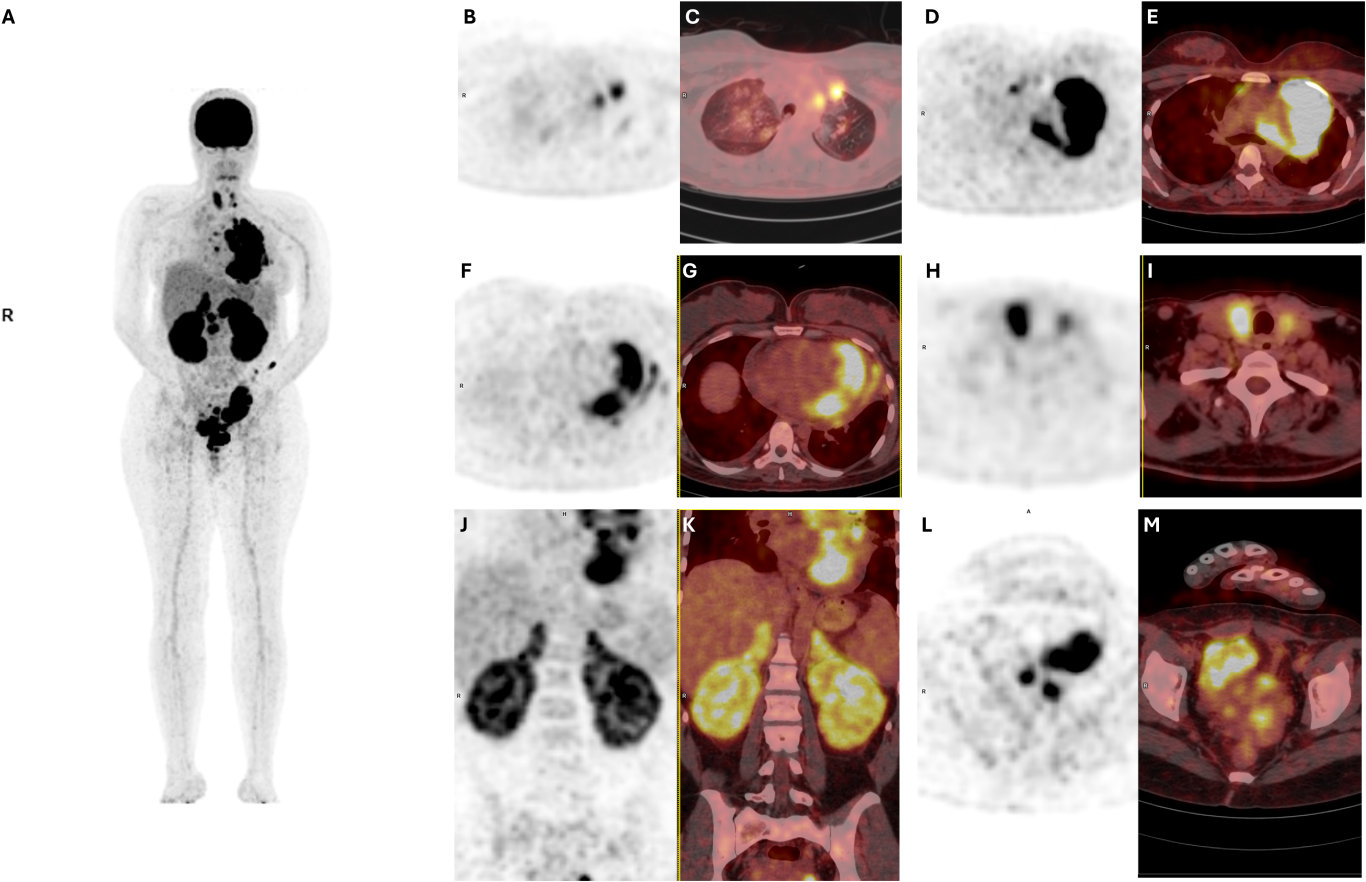
Baseline ^18F-FDG PET and fused PET/CT images obtained prior to treatment demonstrating extensive extranodal disease involvement. **(A)** Whole-body PET maximum intensity projection (MIP) demonstrating extensive metabolically active extranodal disease. **(B, C)** PET and corresponding fused PET/CT images demonstrating intense FDG uptake within bilateral pulmonary parenchymal lesions. **(D, E)** PET and corresponding fused PET/CT images showing FDG-avid supradiaphragmatic extranodal involvement. **(F, G)** PET and corresponding fused PET/CT images demonstrating marked FDG uptake involving the cardiac myocardium, consistent with myocardial infiltration. **(H, I)** PET and corresponding fused PET/CT images demonstrating diffuse thyroid gland hypermetabolism. **(J, K)** PET and corresponding fused PET/CT images demonstrating diffuse hypermetabolic involvement of the kidneys and adrenal glands. **(L, M)** PET and corresponding fused PET/CT images showing increased FDG uptake within the genitourinary organs.

On 8 Nov 2024, Neck ultrasound revealed borderline thyroid enlargement with ill-defined hypoechoic lesions. On 9 Nov 2024, bone marrow biopsy revealed markedly decreased iron stores with no bone marrow infiltration by lymphoma. On 10 Nov 2024, Whole-body diffusion-weighted MRI (**Figure 1F**) confirmed disseminated extranodal involvement, including the ovaries, uterus, kidneys, adrenals, pancreas, spleen, brain, bone, muscle, thyroid, heart, and lung. On 11 Nov 2024, Brain MRI revealed enhancing infiltrative lesions in the right middle cerebellar peduncle, left periventricular area, and septum pellucidum. On 12 Nov 2024, PET scanning (**Figure 2**) revealed hypermetabolic activity within the left ventricular myocardium, pericardial structures, hilar and peribronchial regions, bilateral pulmonary nodules, thyroid, pancreas, kidneys, adnexa, uterus, cervix, vaginal vault, and a solitary marrow deposit at T6.

On 24 November 2024, cytogenetic analysis by fluorescence *in situ* hybridization (FISH) was performed to assess rearrangements involving MYC, BCL2, and BCL6, all of which were negative. In the context of the observed high-grade morphology and a germinal center B-cell immunophenotype (CD10 and BCL6 expression), the absence of MYC and BCL2 rearrangements excludes a diagnosis of double-hit lymphoma (DHL). Although MYC protein expression was present in approximately 40% of tumor cells, the lack of concurrent BCL2 expression and absence of underlying rearrangements argue against a double-expressor phenotype (DEL). Evaluation for 11q aberrations was not performed. Collectively, these findings support a diagnosis of high-grade B-cell lymphoma, not otherwise specified (HGBL-NOS), in accordance with current WHO 2022 and ICC classification criteria (2).

### Staging and differential diagnosis

No lymph node involvement was detected on any imaging modality. Fluorescence *in situ* hybridization (FISH) analysis was negative for BCL6 and MYC rearrangements. Based on the Ann Arbor system, the disease was classified as Stage IV high-grade B-cell lymphoma, NOS due to extensive, disseminated extranodal organ involvement. Differential diagnosis, including metastatic carcinoma, sarcoma, sarcoidosis, and tuberculosis were considered and excluded based on histomorphology and immunophenotypic profile.

### Treatment course

Given the aggressive nature of the disease and cardiac involvement, cytoreductive therapy with R-CEOP was initiated, resulting in improvement of cardiac function and reduced pro-BNP levels. The patient was subsequently transitioned to an intensive alternating R-CODOX-M/R-IVAC regimen (two cycles each), with manageable treatment-related complications (**Table 2**).

**Table 2:** Chemotherapy regimens, observed complications, and clinical management strategies. .

Cycle	Chemotherapy regimen	Main complications/findings	Management
Pre-phase	R-CEOP	Acute pulmonary edema, acute kidney injury, acute liver injury, SVC & left innominate vein non-occlusive thrombosis	Supportive care, heparin therapy
Cycle 1	R-CODOX-M	Acute liver injury, chemical arachnoiditis	Dose reduction (vincristine 50%), dexamethasone, temporary hold, heart failure therapy
Cycle 2	R-IVAC	Acute liver injury, tachycardia	50% dose reduction, leucovorin rescue, antibiotics, supportive care
Cycle 3	R-CODOX-M	No complications	Standard protocol, GnRH agonist protection
Cycle 4	R-IVAC	No complications	Standard protocol, hepatic monitoring

On 13 November 2024, cytoreductive therapy with R-CEOP was administered in the cardiac care unit (CCU) under continuous monitoring due to the risk of ventricular rupture. Treatment was withheld twice due to acute pulmonary edema, acute kidney injury, and acute liver injury, all of which were successfully managed with supportive care. Superior vena cava (SVC) & left innominate vein non-occlusive thrombosis was detected on CT imaging and treated with heparin.

On 26 November 2024, cycle 1 R-CODOX-M was initiated, with dexrazoxane administered before doxorubicin to mitigate cardiotoxicity. Cyclophosphamide on day 5 was withheld due to rising liver enzymes (ALT 540 U/L, AST 55.8 U/L), consistent with acute liver injury (ALI). Chemotherapy was subsequently resumed at 50% vincristine dose reduction. Intrathecal cytarabine, planned for 1 December 2024, was not administered following two unsuccessful attempts. During this cycle, suspected chemical arachnoiditis was treated with dexamethasone.

Follow-up echocardiography revealed regression of myocardial wall thickening with mild left ventricular systolic dysfunction (EF 48%), managed with guideline-directed heart failure therapy (bisoprolol, spironolactone, ramipril).

On 22 December 2024, cycle 2 R-IVAC was initiated and complicated by acute liver injury and tachycardia, managed with 50% dose reduction, leucovorin rescue, antibiotics, and supportive care. Cycle 3 R-CODOX-M (14 January 2025) and cycle 4 R-IVAC (10 February 2025) were subsequently administered without complications, along with gonadotropin-releasing hormone (GnRH) agonist for fertility preservation.

Overall, the patient completed four cycles of alternating R-CODOX-M/R-IVAC over four months with manageable toxicity.

### Outcome and follow-up

**Figure 3:**
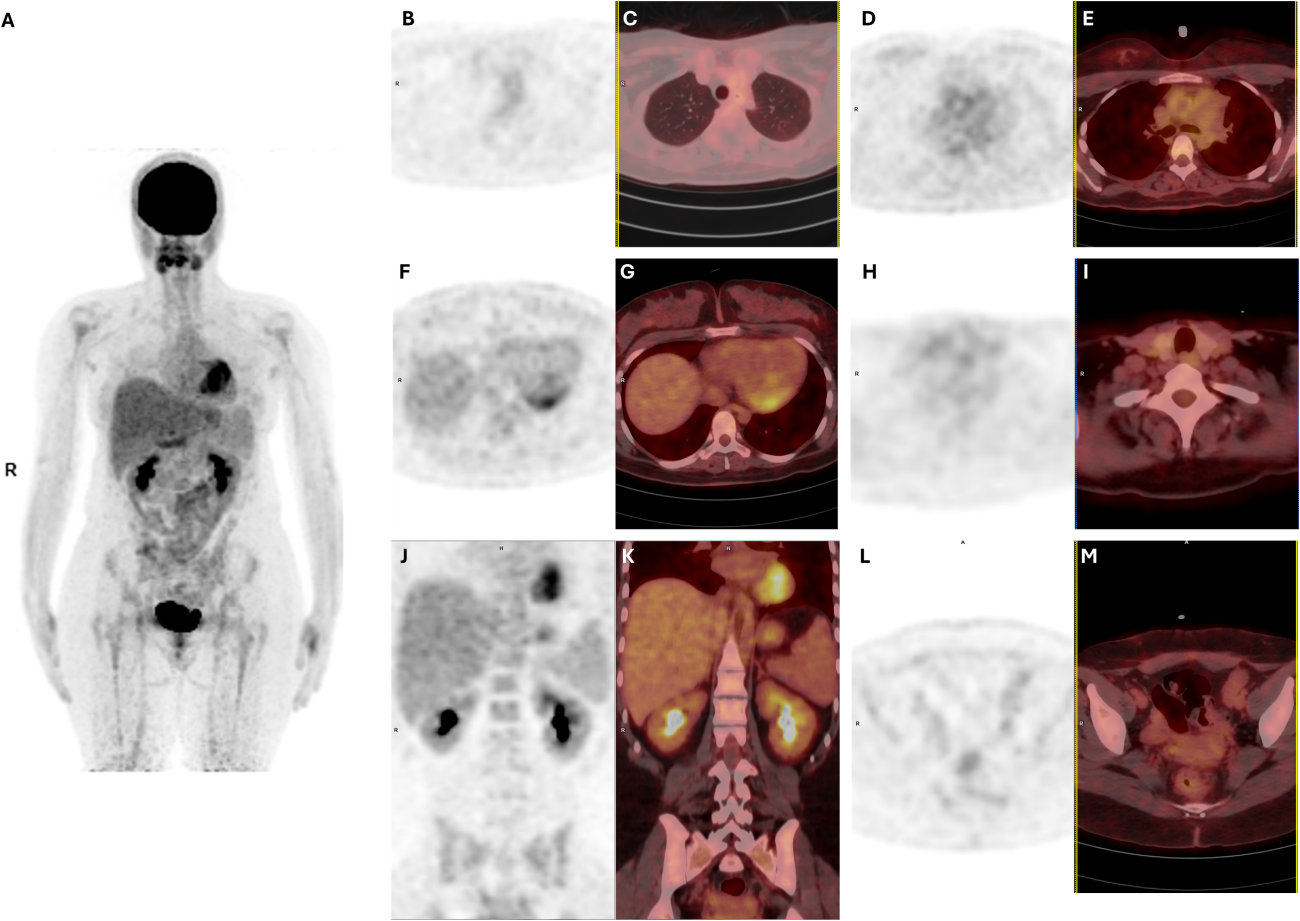
Post-treatment ^18F-FDG PET and fused PET/CT images demonstrating complete metabolic remission. **(A)** Whole-body PET MIP demonstrating resolution of previously observed FDG-avid extranodal disease. **(B, C)** PET and corresponding fused PET/CT images showing complete resolution of previously hypermetabolic pulmonary lesions. **(D, E)** PET and corresponding fused PET/CT images demonstrating absence of abnormal supradiaphragmatic FDG uptake. **(F, G)** PET and corresponding fused PET/CT images showing complete resolution of myocardial FDG uptake. **(H, I)** PET and corresponding fused PET/CT images demonstrating normalization of thyroid gland FDG uptake. **(J, K)** PET and corresponding fused PET/CT images demonstrating normalization of FDG uptake in the kidneys and adrenal glands. **(L, M)** PET and corresponding fused PET/CT images showing resolution of previously increased FDG uptake in the genitourinary organs.

On 23 February 2025, post-treatment follow-up was complicated by febrile neutropenia and severe cytopenias, which resolved with transfusion support and granulocyte colony-stimulating factor therapy. On 3 March 2025, end-of-treatment PET/CT revealed complete metabolic remission (**Figure 3**).

This case report has been prepared in accordance with the CARE guidelines (9).

## Discussion

### HGBL-NOS and diagnostic classification challenges

According to the 5th edition of the World Health Organization (WHO) Classification of Haematolymphoid Tumours (WHO-HAEM5), Tumors harboring concurrent MYC and BCL6 rearrangement with high-grade or large-cell morphology are no longer considered as distinct double hit entity (DHL) as MYC/BCL2. Currently, they are designated as genetic subtypes of HGBL-NOS or diffuse large B-cell lymphoma-not otherwise specified (DLBCL-NOS) (10). HGBL-NOS is defined as heterogenous category of aggressive B-cell lymphomas composed of medium sized or blastoid cells with high-grade morphology, which do not fit into other defined aggressive B-cell lymphomas entities (10).

Overall, under WHO-HAEM5, HGBL are subdivided into three categories: Diffuse large B-cell lymphoma/high-grade B-cell lymphoma with MYC and BCL2 rearrangements, high-grade B-cell lymphoma with 11q aberrations, and high-grade B-cell lymphoma-NOS (2). By contrast, the International Consensus Classification (ICC) divided HGBL into two major categories: HGBL with MYC and BCL2 and/or BCL6 rearrangements, and HGBL-NOS (3). Accurate classification requires integration of cytomorphology, immunohistochemistry (IHC), and fluorescence *in situ* hybridization (FISH). These classification challenges were particularly relevant in this case, given the extensive extranodal involvement in the absence of defining cytogenic rearrangements, which require integration of morphologic, immunophenotypic, and molecular findings.

HGBL-NOS typically affects older adults, with a median age of approximately 70 years and no significant sex predilection (1). Patients often present with high-risk features, including elevated lactate dehydrogenase (LDH), extranodal disease, and central nervous system (CNS) involvement (1). While most non-Hodgkin lymphomas (NHLs) present initially with painless peripheral lymphadenopathy (11), 10-35% of cases are primary extranodal at diagnosis (12). The gastrointestinal tract (GIT) and skin are the most common extranodal sites, followed by testis, bone, and kidney. Less frequently, involvement of the ovary, adrenal glands, heart, thyroid, and bladder has been described (13–19).

This case was highly unusual due to the widespread extranodal disease in the absence of lymph node involvement.

### Diagnostic strategy and role of multimodality imaging

Primary pulmonary lymphoma is rare, accounting for only about 1% of both malignant lymphomas and lung malignancies (20). Clinical manifestations and chest imaging findings are typically non-specific, highlighting the importance of histopathological diagnosis. Bronchoalveolar lavage (BAL) cytology in association with immunohistochemistry and flow cytometry is needed to be considered for diagnosis (21).

In this case, bronchoscopy with endobronchial ultrasound-guided transbronchial biopsy (EBUS-TBNA) confirmed the diagnosis of HGBL-NOS. EBUS-TBNA is a minimally invasive and reliable diagnostic modality for lymphoma, with reported sensitivity of 77%, specificity of 100%, and negative predictive value of 86% when complemented by flow cytometry and immunohistochemistry (22).

Imaging played a central role in disease staging. Positron emission tomography (PET) is highly sensitive and specific for detecting nodal and extranodal NHL, although integrated PET/CT is generally preferred for more precise anatomical localization (23–26). Whole-body diffusion-weighted MRI (DWIBS) is emerging as an alternative staging tool, providing excellent soft tissue resolution and delineation of both nodal and extranodal disease (27, 28). In this case, PET/CT and whole-body MRI demonstrated extensive extranodal infiltration.

Transthoracic echocardiography (TTE) has a reported sensitivity of approximately 60% in the detection of cardiac involvement, and it is useful for functional assessment and treatment monitoring (29–36). However, studies showed that transesophageal echocardiography (TEE) is more sensitive (up to 97%) compared to TTE (31). In this case, cardiac involvement was demonstrated by echocardiography and PET imaging.

### Pathogenesis and molecular features

Tumor cells consistently express pan–B-cell antigens and exhibit a germinal center B-cell-like phenotype (GCB) with CD10 and BCL6 expression in most cases, while MUM1/IRF4 and BCL2 are expressed in approximately 60% and 60-70% of cases respectively. A high proliferative index is a defining feature. Up to 45% of cases carry a single-hit MYC rearrangement, while BCL2 and BCL6 rearrangements occur less commonly (1, 10). Frequently mutated genes include KMT2D and TP53 (37). Gene expression profiling studies suggest overlap with diffuse large B-cell lymphoma (DLBCL), with up to 15% of tumors previously classified as DLBCL reclassified as HGBL (1).

Notably, the Lymphoma/Leukemia Molecular Profiling Project study revealed the poor reproducibility of WHO criteria, with substantial reclassification as DLBCL and BL observed in 53% of 64 reviewed cases (37). Between HGBL-NOS and cases reclassified as DLBCL, there were no significant molecular differences. Among confirmed HGBL-NOS, 57% had germinal center B-cell (GCB), while 25% were activated B-cell tumor (ABC). MYC-R, BCL2, and BCL6 were present in 46%, 10%, and 12% respectively (37).

In a multi-institutional retrospective study of 160 patients with HGBL-NOS, 83% of patients had a GCB, 28% MYC rearrangement, 13% BCL2 rearrangement, and 11% BCL6 rearrangement. Dual-expressor (co-expression of MYC and BCL2) was identified in 37% of these cases, highlighting that DEL occurs across morphologic high-grade B-cell categories, but does not necessarily reflect underlying double-hit genetics (4).

In a retrospective cohort of DLBCL, cases harboring MYC/BCL2 rearrangements (DHL) were strongly enriched within the germinal center B-cell-like (GCB) subtype, with only rare occurrence in ABC/non-GCB tumors (38). High Ki-67 expression, as in this case (90%), is associated with poor prognosis in DLBCL and retains prognostic significance particularly in the rituximab era (39).

### Therapeutic decision-making in advanced HGBL-NOS

Currently, there is no standardized first-line therapy for HGBL-NOS due to the absence of prospective clinical trials. The main clinical dilemma is whether conventional immunochemotherapy with rituximab, cyclophosphamide, doxorubicin, vincristine, and prednisone (R-CHOP) is sufficient, or whether intensified regimens should be used. Evidence suggests that while R-CHOP may be appropriate for rare early-stage HGBL-NOS. In contrast, outcomes in advanced disease and molecular high-grade (MHG) lymphomas are suboptimal, and more aggressive approaches are generally recommended (1). Most MHG lymphomas are of GCB origin, nearly half (49%) harbor MYC-R, and only 36% meet the criteria for DHL. In addition, these tumors are frequently mutated in MYC, BCL2, TP53, KMT2D, and DDX3X (1).

Intensified regimens like R-CODOX-M/IVAC and dose-adjusted etoposide, prednisone, vincristine, cyclophosphamide, doxorubicin, and rituximab (DA-EPOCH-R) protocols have been used as an alternative to R-CHOP (1). R-CODOX- M/IVAC provides CNS penetration through high-dose intravenous methotrexate, essential for treating the CNS involvement in this case (1, 40). The addition of rituximab has improved its efficacy and reduced relapse risk (8, 41, 42). DA-EPOCH-R was excluded given its lack of superiority over R-CHOP in randomized trials (43), and absence of high-dose methotrexate for CNS protection. Although DA-EPOCH-R remains an option for older or less fit patients who cannot tolerate highly intensive regimens (44).

The patient was a candidate for intensive chemotherapy given her young age, good performance status, and high-grade biological features, including MYC overexpression (40%), a markedly elevated Ki-67 index (90%), and GCB origin, which shows superior outcomes in high-risk DLBCL compared to R-CHOP (8). Cardiac infiltration required initial R-CEOP cytoreduction to stabilize cardiac function before escalating to the full protocol (32). R-CEOP cytoreduction was followed by the intensified R-CODOX-M/IVAC regimen. This approach was selected to provide systemic disease control while ensuring CNS prophylaxis.

The frequent downregulation of MHC class 2 expression and immune response pathways observed in MHG lymphomas provide a biological rational for the emerging role of immune-based therapies, including directed chimeric antigen receptor T-cell therapy (CAR-T) and CD20/CD3-bispecific antibodies (1).

Several newer treatments are being studied for relapsed or refractory HGBL. CD19- directed CAR-T cell therapies, including axicabtagene ciloleucel (axi-cel) and tisagenlecleucel (tisa-cel), have produced objective response rates exceeding 50% in relapsed/refractory DLBCL, with complete remissions maintained in 30-40% of patients (45, 46). Lisocabtagene maraleucel (liso-cel) is associated with less cytokine release syndrome and neurotoxicity compared to earlier CAR-T products (47). Although data specific to HGBL-NOS is limited, CAR-T therapy offers an alternative for patients whose disease is refractory to salvage chemotherapy or who relapse despite intensive induction (48).

Frontline treatment approaches are also evolving. The BCL-2 inhibitor venetoclax combined with R-CHOP showed improved complete response rates in BCL2-positive subgroups in the phase 2 CAVALLI study (49). Polatuzumab vedotin, an antibody-drug conjugate that target CD79b, demonstrated superior 2-year progression-free survival when combined with R-CHP comparted with R-CHOP in intermediate and high-risk DLBCL (50). The bispecific antibodies glofitamab and epcoritamab, which target both CD20 and CD3, have demonstrated complete response rate of 35-39% in relapsed or refractory large B-cell lymphoma (51, 52).

Despite these advances, HGBL-NOS remains difficult to classify at the molecular level, which limits the ability to tailor therapy. Future studies examining gene expression patterns and recurrent mutations are needed to identify which patients will benefit most from these specific treatments (37).

### Prognostic considerations

The prognosis of HGBL-NOS is generally inferior to DLBCL, NOS, but more favorable than that of HGBL with double- or triple-hit genetics (53–56). Currently, no reliable prognostic markers have been validated, although patients with lower International Prognostic Index (IPI) scores (0–2) appear to fare better (55, 57, 58).

## Conclusion

This case highlights an exceptionally rare and aggressive presentation of HGBL-NOS, characterized by extensive extranodal organ involvement in the absence of lymphadenopathy. It emphasizes the diagnostic and therapeutic challenges, prompting an integrated, multidisciplinary approach involving advanced imaging modalities, histopathology, immunophenotyping, molecular studies, and optimized chemotherapy protocols. As a single-patient observation, these findings may not be generalizable. Further studies are warranted to determine whether a conventional or intensified chemotherapy regimen is most appropriate. This case report emphasizes the importance of early recognition and tailored intensive therapy in achieving remission in rare HGBL-NOS presentations.

## Data Availability

The original contributions presented in the study are included in the article/supplementary material. Further inquiries can be directed to the corresponding authors.
